# Ambulatory oxygen: why do COPD patients not use their portable systems as prescribed? A qualitative study

**DOI:** 10.1186/1471-2466-11-9

**Published:** 2011-02-11

**Authors:** Elizabeth Arnold, Anne Bruton, Maggie Donovan-Hall, Angela Fenwick, Bridget Dibb, Elizabeth Walker

**Affiliations:** 1 School of Health Sciences, Highfield Campus, University of Southampton, Hampshire, UK; 2 Portsmouth Community PCT, The Turner Centre, St James' Hospital, Portsmouth, Hampshire, UK; 3 School of Medicine, Highfield Campus, University of Southampton, Hampshire, UK; 4 School of Social Sciences, Brunel University, Uxbridge, Middlesex UK; 5 Respiratory Centre, Queen Alexandra Hospital, Cosham, Portsmouth, Hampshire, UK

## Abstract

**Background:**

Patients with COPD on long term oxygen therapy frequently do not adhere to their prescription, and they frequently do not use their ambulatory oxygen systems as intended. Reasons for this lack of adherence are not known. The aim of this study was to obtain in-depth information about perceptions and use of prescribed ambulatory oxygen systems from patients with COPD to inform ambulatory oxygen design, prescription and management.

**Methods:**

A qualitative design was used, involving semi-structured face-to-face interviews informed by a grounded theory approach. Twenty-seven UK community-dwelling COPD patients using NHS prescribed ambulatory systems were recruited. Ambulatory oxygen systems comprised cylinders weighing 3.4 kg, a shoulder bag and nasal cannulae.

**Results:**

Participants reported that they: received no instruction on how to use ambulatory oxygen; were uncertain of the benefits; were afraid the system would run out while they were using it (due to lack of confidence in the cylinder gauge); were embarrassed at being seen with the system in public; and were unable to carry the system because of the cylinder weight. The essential role of carers was also highlighted, as participants with no immediate carers did not use ambulatory oxygen outside the house.

**Conclusions:**

These participants highlighted previously unreported problems that prevented them from using ambulatory oxygen as prescribed. Our novel findings point to: concerns with the lack of specific information provision; the perceived unreliability of the oxygen system; important carer issues surrounding managing and using ambulatory oxygen equipment. All of these issues, as well as previously reported problems with system weight and patient embarrassment, should be addressed to improve adherence to ambulatory oxygen prescription and enhance the physical and social benefits of maintaining mobility in this patient group. Increased user involvement in both system development and service provision planning, could have avoided many of the difficulties highlighted by this study.

## Background

The need for user involvement in healthcare research is now well established in the UK National Health Service (NHS)[1]. This concept is also becoming more common in new medical device development through projects such as the Multidisciplinary Assessment of Technology Centre for Healthcare (MATCH)[2]. However, many devices currently in use within the NHS were designed prior to the introduction of this concept, including oxygen delivery systems. This paper explores reasons for non-use of a specific health technology and highlights the necessity for research to tease out the needs of patients and involve them in designing new devices and systems.

Ambulatory oxygen (AO) provides a way to allow patients with severe COPD to continue to remain active within their community, maintaining exercise tolerance and social interaction. In the UK, AO became widely available for prescription in 2006[3]. Under British Thoracic Society guidelines prescription of AO for patients with COPD should follow specific criteria either as an addition to long-term oxygen therapy (LTOT) or for exercise desaturation in patients who are normoxic at rest. However, in some regions of the UK patients continue to be prescribed LTOT and AO by a General Practitioner (GP), or after discharge from hospital, with no formal assessments and no follow-up. It is known that patients with COPD frequently fail to adhere to their oxygen prescription[4]. A trial specifically investigating the use of AO[5] was prematurely ended because participants were not using their AO systems. This situation is clearly wasteful of health resources.

Adherence to prescribed medication is known to be fundamental to optimising the management of long-term conditions[6]. However, despite recent improvements in COPD management, high levels of non-adherence remain and have been shown to be predominantly influenced by patient beliefs, behaviours and experiences[7]. Croxton and Bailey have suggested that the underuse of AO, and the reasons for this underuse, need further research[8]. These authors also advocate research to clarify the benefits of AO, and the way that patients with COPD use AO, as a priority for medical researchers. This call for more research into the use of AO has been echoed by others, including the 2010 Department of Health consultation document on COPD services in England[9-12]. Albert and Calverley have suggested that whatever clinical benefits patients with COPD may derive from using AO, what remains largely unknown is how these patients use AO in their day to day lives[11].

There is little published evidence on why COPD patients choose to use their AO cylinders or not. Without this information it will be difficult to support patients who may have difficulty using AO, and ensure that active patients receive optimal levels of AO. This qualitative study therefore aimed to explore patients' use of AO, by asking patients with COPD who had already been prescribed AO, to identify and describe what influenced their use of their AO system within the community.

## Methods

The objective of this study was to gain information from patients themselves. A questionnaire approach was rejected because it was important to understand the reasons patients had for using or not using their AO and the perceptions underlying this behaviour. Therefore an inductive qualitative methodology was employed using a semi-structured interview guide. Grounded Theory (GT) was chosen as the methodological approach because GT aims to produce theory about the area under study[13]. GT as described by Glaser and Strauss in 1967, uses a method of constant comparative analysis for analysing qualitative data to produce relevant social theory inductively[14]. Each interview is transcribed and analysed before the next interview so that areas of interest which have been voiced by previous participants can be explored further. As a study continues, more specific participants are recruited to the study to strengthen or refute any emergent theories, such as differences in perceptions of participants with or without carers, living in a family or living alone. This study was underpinned by an objectivist philosophy and placed the researcher as a detached collector of objective data. The GT approach in this study was adapted from its purest form because some of the tenets of the original approach (no prior knowledge; theoretical sampling) are incompatible with conducting ethical NHS research. A literature search preceded Ethical submission for this study, and the semi-structured questionnaire was based partly on this literature search and partly on researchers' clinical experience. In line with a GT approach, further literature searches occurred as theory began to emerge inductively from the study findings. This study also involved second interviews approximately one year after the original interviews (with a sample of the original participants) to ensure the study results accurately reflected the views of the participants; this acted as a means to increase the validation and credibility of the reported results. Full ethical approval was obtained from the Local Research Ethics Committee (Isle of Wight, Portsmouth and South East Hampshire REC 06/Q1701/61) prior to recruitment.

### Recruitment and sampling

#### Inclusion criteria

• Patients registered at the Respiratory Centre, Portsmouth, UK

• Documented diagnosis of COPD

• Prescription for ambulatory oxygen

#### Exclusion criteria

• Additional co-morbidities that may have affected oxygen usage

• Inability to take part in interviews due to cognitive or language difficulties

• Living outside the area covered by the Local Research Ethics Committee

• Recent (within three months of interview) admission to hospital

Patients who met the inclusion criteria were identified by a respiratory nurse from a register at the Respiratory Centre responsible for oxygen assessments. Potential participants were posted an invitation letter by the nurse and asked to reply if they wished to be included in the study. In line with qualitative interviewing principles, as wide a variety of individuals as possible were approached for recruitment (that is, purposive sampling was used to gain a diversity of opinion), irrespective of age, gender, living arrangements or length of time with AO prescription. Forty-four invitations were posted, from which 34 responded positively. One of these died prior to interview, two decided not to be interviewed, and five were excluded for not meeting the entry criteria.

### Interviews

Twenty-seven individuals with COPD agreed to be interviewed in their own homes, where written informed consent was obtained. The researcher used a semi-structured interview topic guide (see Additional file 1). This was initially devised from the researcher's clinical experience, but transcripts were analysed after each interview (prior to the next interview). This enabled the researcher to alter the topic guide to incorporate themes as they emerged and use them at subsequent interviews. This process continued until saturation was reached and no new information was forthcoming. In line with the objectivist GT approach to this study, the interviewer attempted to remain detached from interaction with the participant during the interview process, apart from encouraging the participant to expand on answers and to clarify any unclear statements. All interviews were audiotaped with permission. Carers were frequently present during interviews and their comments were recorded and included with permission. Respondent validation interviews took place from six to 12 months after the initial interviews, with seven of the original participants (representing 33% of those still alive).

### Data Analysis

All interview recordings were transcribed and anonymised by one of the authors (EA). The transcripts were analysed initially using open coding where each line of the transcript was analysed to identify data pertinent to the research question and relevant data were recorded under a heading or code. Open codes were compared for similarities and differences using constant comparative analysis, and links between codes and categories formed. Information derived from early participants was used to frame areas for discussion with subsequent participants. New information relating to the lack of information available, fear of running out, and carer support was derived in this way. Possible emergent theory, derived by examining categories, was established by using 'common sense' linkages within the data. Negative cases (views expressed that differed from the majority) were also identified. Data collection continued until no new findings emerged from the data analysis and saturation was deemed to have occurred. Extensive memo writing accompanied all stages of the analysis. A second experienced qualitative researcher independently produced codes for a sample of the participants, to verify the analysis procedure. No divergence in the two analyses was found, although the second analyser suggested additional areas of coding and emphasised supporting or conflicting data, or areas needing more investigation during the interviews. The subsequent respondent validation interviews with participants supported the original data, emergent coding and main findings of the study.

## Results

Of the 27 participants, 14 were male (50%) and 24 were over 60 years of age (88%). The average age was 68 years, with a range from 54 to 85 years. No formal testing of clinical status was undertaken as it was not relevant to the research question. Twenty-three (85%) participants were married with their spouse as the primary carer. Four (15%) lived alone and relied on relatives, who lived some distance away, for any support. Transport: Seven (26%) of the 27 participants had given up driving and were reliant on electric buggies to take them to the local shops or to appointments; four (15%) did not have any personal transport available to them; one used a motorcycle; and the remaining 16 (59%) had access to a car. Oxygen: Two of the participants (7%) did not qualify for long term oxygen therapy (LTOT) and had stand alone AO cylinders; eleven (41%) already used LTOT before being prescribed AO; the remaining 14 (52%) received both LTOT and AO together. All participants were prescribed an oxygen cylinder weighing 3.4 kg which was delivered with a shoulder bag to allow the patient to carry the cylinder. All participants had their AO cylinders delivered by the local provider and used nasal cannulae to interface with the AO system.

### Main findings

The main barriers to AO use were strongly felt by most of the participants, so that codes were saturated very quickly for those areas. The subsequent respondent validation interviews supported the original data and emergent coding. The selected quotations have been chosen as the words or phrases which best represent (in the researchers' opinion) all the patients' views in the domain described. Negative cases are described after the majority view has been expressed.

The participant (Pt) number, the page and line of the transcript are in brackets after each quotation. Carer comments are identified as 'Carer'.

#### 1. Lack of information: confusion around AO use

Twenty-five participants could not recall receiving any information on the use of their AO system from a healthcare professional. The two who could recall such information had received their AO from their Respiratory Centre. No-one who had received AO as part of a discharge package from their local general hospital or from their GP, reported receiving any AO instructions from professionals.

*"They wouldn't let me home *(from hospital) *until that concentrator was installed and that *(AO) *came with it. We got a booklet for the concentrator but the guy who brought it *(AO) *told me to turn it on to two litres a minute, he said turn it to that so that's how we use it." *(Pt5, 5, 145)

*"I asked for it *(AO) *and got it through the GP, I was very breathless when I went out of the house and couldn't cope so I asked him for it, but that was it, I don't remember any instructions just use it when I felt I needed it and that was on an ad hoc basis." *(Pt1, 3, 71)

There were no negative cases reported in this domain.

#### 2. Lack of benefit: the AO system makes no difference

Thirteen participants described perceiving a lack of benefit from the AO in two different ways:

a) When they used AO to relieve their breathlessness, it did not make them feel better, or stop them getting breathless.

b) When going out they were usually sitting on an electric buggy, or wheelchair, and therefore not exerting themselves. Thus they did not feel more breathless, so felt no need to take their AO system out with them.

*"Getting no benefit out of it at all, I don't know that they (AO cylinders) are making any difference to my breathing, because I'm still out of breath, and that's the problem... I don't think it's helping me, we don't understand why I'm still out of breath"*. (Pt9, 5, 150)

It is not known how participants derived their expectations of oxygen as a means of relieving breathlessness, but even those who had been prescribed long term oxygen for 24 hours per day, did not take AO out with them.

Twelve of the participants expressed deriving some benefit from AO but this was related primarily to the presence of the AO cylinders providing increased feelings of security when outside the home (even when not in use), rather than relief of breathlessness, or having increased exercise capacity.

#### 3. Fear: the AO might run out

Cylinders were equipped with a small clock-face dial to indicate the amount of remaining oxygen using a traffic light system: green for full (to a half), orange (for one half-to one quarter full) and red for a quarter full. Practically, this dial was obscured by the jacket covering the cylinder, so users had to lift the cylinder out of the jacket to look at the gauge. Eleven participants expressed a fear of the cylinder running out of oxygen when they were away from home.

*"I'm worried it's (AO) going to run out, I can see the dial going down, so I don't like to go too far away from this one *(long term oxygen therapy system)*, in fact it restricts me in some ways because I can't go out for long." *(Pt 13, 5, 137)

Those participants who did not feel that reliability was a problem were those who had never experienced a cylinder running out when outside the home.

#### 4. Carers: needing help to use AO outside the house

Only one participant in this sample was able to carry and use the AO system in the manner envisaged by prescribing health care professionals, which is to take them out and about in public independently. Carers and personal transport were fundamental to all other participants who wished to take AO out of the house with them. Carers carried the AO to and from the car, or on and off the electric buggies, and also checked the cylinder gauges.

Carer*: "She doesn't think it's a problem the weight because she doesn't carry it, I put it in the car between us and she uses it as she needs to when we're going along. I sort out all the oxygen, we have one in the car and this one in the house is a back-up. I have to check them all the time 'cause she leaves them on" *(Pt 3, 8,237)

Four participants in this study had no immediate carer and relied on relatives, who lived some distance away, to help them go out. Some of these relatives had difficulties themselves, which precluded them from helping with an AO system.

*"Well I only go out if my daughter is with me, it's *(AO) *too heavy to use to carry. We get a taxi...my daughter would have to carry it from the taxi to the wheelchair but she has been unwell and it's too much for her." *(Pt15, 4, 117)

These four participants left the house infrequently. The absence of an immediate carer meant they had no means to carry the AO system, and they were unable to use it outside the home. The one negative case in this domain is explained at the beginning of this section.

#### 5. Embarrassment: not wishing to be seen using AO

In this sample, embarrassment was a very significant factor affecting behaviour. Twenty-one participants complained that they disliked being seen out with an AO cylinder. Even participants on 24 hour supplementary oxygen did not use their AO out in public. Feelings of embarrassment resulted in some counter-intuitive use of the AO system. For example, several participants reported using the AO on a car journey to their destination (for example to shops, or social events) then leaving the AO in the car while out in public (for example, in the shop), before resuming the AO use when returning to the car.

"... *it's just that I feel that people would stare at you and it would make me feel very uncomfortable" *(Pt25, 4, 109)

"*I wouldn't like people looking at me, I just feel embarrassed about it...I think it's probably me, I have always been very independent and I don't like being ill...and I don't want other people to know I'm ill" *(Pt2, 3, 89)

This was true even for those who had a willing carer to carry the equipment. Of the participants who were using AO out of the house, only two did not feel embarrassed at using AO in public.

#### 6. Weight: AO is too heavy

Only one participant was carrying his oxygen system out in the community. Twenty-five of the 27 participants reported that the weight of the cylinder was a barrier to use. Participants suggested that carrying the cylinder would impact negatively on their ability to walk anywhere, and made it more difficult to go outside.

*"I mean how on earth are you supposed to lug a great weight like that around and walk very far, this is my problem I can walk in here (*home*) but I can't walk very far when I'm out because I'm lugging that, it's ridiculous, I have to have someone with me just so I can take that cylinder." *(Pt27, 3, 96)

*"If they could invent something lighter and smaller I'd give it a go but it's totally unsuitable for the way I live my life." *(Pt 20, 4, 217)

There was only one negative case in this domain, described at the beginning of this section.

## Discussion

The main purpose behind ambulatory oxygen provision is to enable people with COPD to remain mobile and socially active, while preventing oxygen desaturation. This research found that problems with the equipment itself prevented this outcome. Additionally, lack of information, stigma and lack of social support all contributed to the difficulties patients encountered when trying to use this prescribed medication. One of the most interesting findings was the dissonance between what health professionals believe patients are doing, and the reality on the ground, which was discovered through an in-depth qualitative approach within the home environment. It has been argued that non-adherence has less to do with patient culpability and more to do with failure to consider individuals' circumstances at the time of prescription[15]. In this study, inappropriate ambulatory equipment had been prescribed to COPD patients, many of whom were physically unable to use the system. Cylinders were too heavy and cumbersome to carry, and even if carers were present to assist, they often had their own medical problems which made using AO virtually impossible. Many participants still enjoyed active social lives, but found it daunting to use their AO systems in public, as they feared being perceived as 'different' or an 'invalid'. The majority solved this dilemma by not using their AO.

The importance of providing specific information to allow patients to self-manage a chronic condition is well documented[16]. Patients negotiate their use of medicines in the light of personal need and their daily routines, and ultimately decide what is right for them; but education is essential to enable informed decisions. There is little published literature relating to specific medical instructions on the use of AO, although Petty and Bliss state that desaturating patients should use AO 'whilst walking'[17]. In our study no participants could recall receiving specific instructions on AO usage from health professionals, and so followed a generic instruction to "use it when you're out". The lack of specific instructions found within this study may reflect a wider uncertainty within the medical profession as to the optimum prescription of oxygen through a 24 hour period. Current prescribing is based on trials conducted 30 years ago[18], the conclusions from which have been questioned[8]. Guidelines for patient information provision on AO are not currently available. Professionals need to decide if allowing oxygen delivery personnel to give patients instructions on the use of AO is a desirable consequence of not giving more specific written personalised instructions to AO patients.

In this study those participants who felt they derived no benefit from AO, did not attempt to use it outside. Non-adherence to medication because no benefit is perceived has been reported in research into the use of general medication by COPD patients[7,19,20]. Perceptions of benefit are related to expectation, but it is difficult to establish where and how patients build their expectations. The provision of specific AO information would reduce the development of unrealistic expectations, and should thereby increase perceptions of benefit and encourage adherence. Alternatively, lack of perceived benefit may result from inappropriate prescription (in this sample not all prescriptions followed assessment in secondary care for specific exertional desaturation), or through incorrect use of AO.

Fear of the oxygen running out has not been previously reported in AO research. Technical equipment is the only way oxygen can be delivered to a patient, and lack of confidence in the equipment reduced willingness to go out of the house with the system. A study of 65 diabetic patients has suggested that lack of confidence in insulin-delivery devices compromised adherence to medication[21]. In this study, those participants who thought the gauge was unreliable, and that the cylinder might run out, perceived this to be a barrier to AO use. Robust research data on the reliability and accuracy of oxygen gauges are not available, but anecdotal clinical information suggests less than optimal accuracy, so there may be some basis for these participants' fears.

Embarrassment was a common theme, confirming findings from a small qualitative study by Williams et al who reported that COPD patients described feelings of embarrassment because of the visibility of their oxygen equipment[22]. Earnest (2002) also reported that patients who used oxygen described embarrassment and self-consciousness when using AO[23]. COPD has very visual side effects i.e. the patient is seen to be breathless, coughs aloud, and may need to carry overt medication (such as AO, metered dose inhalers, or nebulisers) with them. This marks the patient out as someone who is different from the rest of the population and has stigmatising consequences[24]. Participants in this study voiced very strong opinions about not wanting to be different, or stand out from the crowd and this influenced their use of AO outside the home. To date there has been very little research into stigma in COPD, and how it may affect behaviour. Social support was essential in overcoming feelings of embarrassment, and in enabling participants to overcome the physical barriers to use of their AO (that is, to carry and maintain the equipment). Those participants who had no carers and no transport never used their AO outside the house. Previous research has suggested that positive social support has a direct influence on self-care activities and coping skills in COPD[25], but this is the first description of the essential role played by carers to enable patients with COPD to use their AO appropriately.

The negative impact of the weight of AO equipment on use has been documented by previous researchers[4]. One study looking at adherence levels in long term oxygen therapy for a mixed pathology group found that 58% of 192 participants were not using the AO outside the house, with system weight cited as the most important factor affecting usage[26]. Several studies suggest that muscle weakness in COPD is a significant co-morbidity[27] and that therapeutic interventions such as long-term steroid use may contribute to this muscle weakness[28]. Most of these study participants were over 65 years of age, and had COPD for several years, which may partly explain their inability to carry the AO system.

### Limitations

Participants in this study all used one type of cylinder AO, so these findings cannot be generalised to patients using other types of AO, although clinical observation indicates that many of the issues raised are similar for patients using liquid oxygen. All participants were recruited from one geographical area in the South of the UK, although there were different AO prescribers within this area.

### Implications for future research and clinical practice

If AO is going to fulfil its potential to keep COPD patients more physically and socially mobile, then patients require detailed, personalised plans of how and when to use it. Any desaturating COPD patient should be given specific instructions on use of AO during activity. Patients who feel information about medication is personal to them are more likely to use that medication[29]. The effect of such specific information provision on subsequent AO use in the community is an important area for future research. Patients need help to find ways of managing the perceived barriers, particularly if they have no carers, or have carers who are unable to help them. Patients also need help to overcome the embarrassment they feel when using AO out of the house. This might require psychological support, or more information as to why they need to use AO. Patients need more information on why maintaining their exercise levels is important to their disease process, even at this supplementary oxygen stage of the COPD pathway. The NHS standard AO equipment itself could also be improved, in terms of reducing weight and increasing portability, reducing its overt 'medical' properties (to reduce stigmatisation), and increasing the reliability of its gauges.

## Conclusions

Grounded theory is designed to produce theory about the area under study. The theory produced as a result of this research is that COPD patients who perceive that AO negatively adds to the physical, mental or social burden of their condition are unlikely to use their system. COPD patients with the NHS standard type of AO system are unable to use their medication as prescribed because of the difficulties they experience with the AO system itself i.e. principally the perceived unreliability of the gauges and the weight of the system. There is also considerable embarrassment felt at being seen with an AO system in public. This highlights the difficulties that patients face when asked to incorporate medical technology into their lives and illustrates the pitfalls associated with designing technologies without reference to the ultimate end users. Novel findings in this study were the lack of specific information provision for those prescribed AO, the fear that the oxygen might run out (perceived gauge unreliability), and the essential role of the patients' carers in managing and maintaining the AO equipment. It is debatable which of these issues has greater significance in terms of affecting AO use, but all of them could be addressed with appropriately designed interventions involving patient input to improve the acceptability of existing equipment, to design better equipment, and to improve the quality of the information provided to patients and carers. Attention to each of these issues may be needed to optimise adherence to AO prescription and enhance the physical and social benefits of maintaining mobility in this patient group.

## Competing interests

(1) AB and LA have worked on a related UK Dept of Health (HTD programme) funded project in collaboration with Luxfer Gas Cylinders Ltd. However, Luxfer Gas Cylinders Ltd played no role in the design, data collection, analysis, interpretation or writing up of this study. LA, MD-H and AF are employees of the University of Southampton which received grant reference: HTD352; (2) MD-H, AF, BD and EW have no relationship with any company that might have an interest in the submitted work in the previous 3 years; (3) their spouses, partners, or children have no financial relationships that may be relevant to the submitted work; and (4) all authors have no non-financial interests that may be relevant to the submitted work

## Authors' contributions

AB had the original idea for the overall protocol and contributed to the funding application. All authors contributed to the development of this part of the protocol and the overall supervision and conduct of the study. LA conducted the interviews and led the qualitative analysis with input from MD-H, AF and BD. EW participated in the design and conduct of the study. LA and AB drafted the paper. All authors contributed to the writing of the final version of the paper.

## Pre-publication history

The pre-publication history for this paper can be accessed here:

http://www.biomedcentral.com/1471-2466/11/9/prepub

## Supplementary Material

Additional file 1 **Semi-structured interview schedule**. Examples of questions used during the interviews with participants Click here for file
