# Supplementary material for: Ambulatory oxygen: why do COPD patients not use their portable systems as prescribed? A qualitative study
Source: BMC Pulm Med. 2011 Feb 11;11:9. doi: 10.1186/1471-2466-11-9 (PMC3045998; doi:10.1186/1471-2466-11-9)
Supplement: Additional file 1 — Semi-structured interview schedule. Examples of questions used during the interviews with participants [file 1471-2466-11-9-S1.DOC]

**Ambulatory oxygen: why do COPD patients not use their portable systems as prescribed? A qualitative study.**

Additional file 1

## Semi-structured interview schedule

Examples of questions used during the interviews with participants

Can you tell me how long you have had a problem with your chest?

- When did you get the diagnosis? (probe: who diagnosed COPD?)

How does your chest condition affect you? (probe: daily life? particular problems?)

Do you have oxygen at home, how much do you use that?

- How do you use oxygen during the day/night?

How do you cope around the house?

How often do you leave the house?

- Where do you go?
- How do you manage when you leave the house?
- Do you take your AO system with you (probe: if not why not?)
- How do you manage your AO outside the house?

What do you think about your AO system?

- How useful is it to you? (probe: if useful why; if not useful, why not?)

Who told you how to use the system? (probe: what did they say?)

Do you feel comfortable using your AO system? (probe: if so why; if not why not?)
